# *Listeria monocytogenes* Alters Mast Cell Phenotype, Mediator and Osteopontin Secretion in a Listeriolysin-Dependent Manner

**DOI:** 10.1371/journal.pone.0057102

**Published:** 2013-02-27

**Authors:** Catherine E. Jobbings, Hilary Sandig, Jayde K. Whittingham-Dowd, Ian S. Roberts, Silvia Bulfone-Paus

**Affiliations:** 1 Faculty of Human and Medical Sciences, University of Manchester, Manchester, United Kingdom; 2 Faculty of Life Sciences, University of Manchester, Manchester, United Kingdom; 3 Research Center Borstel, Borstel, Germany; Institut National de la Santé et de la Recherche Médicale U 872, France

## Abstract

Whilst mast cells participate in the immune defence against the intracellular bacterium *Listeria monocytogenes*, there is conflicting evidence regarding the ability of *L. monocytogenes* to infect mast cells. It is known that the pore-forming toxin listeriolysin (LLO) is important for mast cell activation, degranulation and the release of pro-inflammatory cytokines. Mast cells, however, are a potential source of a wide range of cytokines, chemokines and other mediators including osteopontin, which contributes to the clearing of *L. monocytogenes* infections *in vivo*, although its source is unknown. We therefore aimed to resolve the controversy of mast cell infection by *L. monocytogenes* and investigated the extent of mediator release in response to the bacterium. In this paper we show that the infection of bone marrow-derived mast cells by *L. monocytogenes* is inefficient and LLO-independent. LLO, however, is required for calcium-independent mast cell degranulation as well as for the transient and selective downregulation of cell surface CD117 (c-kit) on mast cells. We demonstrate that in addition to the key pro-inflammatory cytokines TNF-α and IL-6, mast cells release a wide range of other mediators in response to *L. monocytogenes*. Osteopontin, IL-2, IL-4, IL-13 and granulocyte macrophage colony-stimulating factor (GM-CSF), and chemokines including CCL2, CCL3, CCL4 and CCL5 are released in a MyD88-dependent manner. The wide range of mediators released by mast cells in response to *L. monocytogenes* may play an important role in the recruitment and activation of a variety of immune cells *in vivo*. The cocktail of mediators, however, is unlikely to skew the immune response to a particular effector response. We propose that mast cells provide a hitherto unreported source of osteopontin, and may provide an important role in co-ordinating the immune response during *Listeria* infection.

## Introduction

Mast cells are traditionally associated with the immune response against multi-cellular parasites and the pathogenesis of allergy and asthma, although there is increasing evidence that they are important in immunity to pathogenic bacteria [1]. They are strategically located at the interface between the host and the environment, including the gut, allowing direct interaction with bacteria and bacterial products [1], [2]. There is increasing evidence of the importance of mast cells during infection with the intracellular pathogen *Listeria monocytogenes* [3].

*L. monocytogenes* is a Gram-positive food-borne pathogen which predominantly affects the immunocompromised, pregnant women and newborns [4], [5]. Mast cells are crucial for clearance of *L. monocytogenes in vivo,* with mast cell-depleted mice having significantly more viable bacteria in the liver, spleen and peritoneum after infection [3]. The importance of mast cells has been attributed to their degranulation in response to the bacterial toxin listeriolysin (LLO). During degranulation, pre-formed tumour necrosis factor-α (TNF-α) is released resulting in the recruitment and activation of neutrophils [3], [6]. TNF-α, however, is just one of the cytokines released in response to *L. monocytogenes* with other pro-inflammatory cytokines, including IL-6 and IL-1β, released during infection *in vivo* [7], [8], and mast cells have been shown to secrete these in response to *L. monocytogenes in vitro* [7], [9], [10].

Osteopontin (OPN) is an *O-*glycosylated phosphoprotein which is secreted by a wide range of cells and has a protective role in immunity to some bacterial pathogens [11]. OPN has been shown to be required for the clearance of *L. monocytogenes* after systemic infection [12] although a more recent study did not replicate this finding [13]. Although T cells are known to produce OPN during bacterial infection [14], the source of OPN in *L. monocytogenes* infections is unknown. Mast cells release OPN after stimulation with antigen and a range of toll-like receptor (TLR) agonists [15], so could be a potential source.

Due to their location, mast cells may directly contact *L. monocytogenes* and could potentially be infected. There is, however, contradictory evidence regarding mast cell infection by *L. monocytogenes in vivo* and *in vitro.* Peritoneal mast cells from mice infected with *L. monocytogenes* contain viable bacteria [3], [16] and infection of bone marrow-derived mast cells (BMMC) *in vitro* has been reported [17]. Other studies, however, have been unable to identify intracellular bacteria in BMMC *in vitro* [3], [16], and the importance of the pore-forming toxin LLO for mast cell infection has not been investigated.

Most of the work on mast cells during *L. monocytogenes* infections has been performed using a murine model following intraperitoneal or intravenous injection of bacteria. As *L. monocytogenes* is a food-borne pathogen and the main route of infection is oral, the importance of these responses is unclear. Furthermore, it is difficult to delineate the specific role of mast cells in a whole organism. The aim of this study was to address to the controversy of mast cell infection by *L. monocytogenes* and to investigate the extent of mediator release from mast cells.

We confirm that mast cells are infected, albeit inefficiently, by *L. monocytogenes.* We find that in addition to degranulation and release of pre-formed pro-inflammatory mediators, mast cells also synthesise and secrete OPN and a variety of cytokines and chemokines in response to infection by *L. monocytogenes*. Mast cells, therefore, have the potential to co-ordinate a wide range of leukocytes through their release of mediators during *L. monocytogenes* infections.

## Results

### *Listeria monocytogenes* infects and survives within mast cells

**Figure 1:**
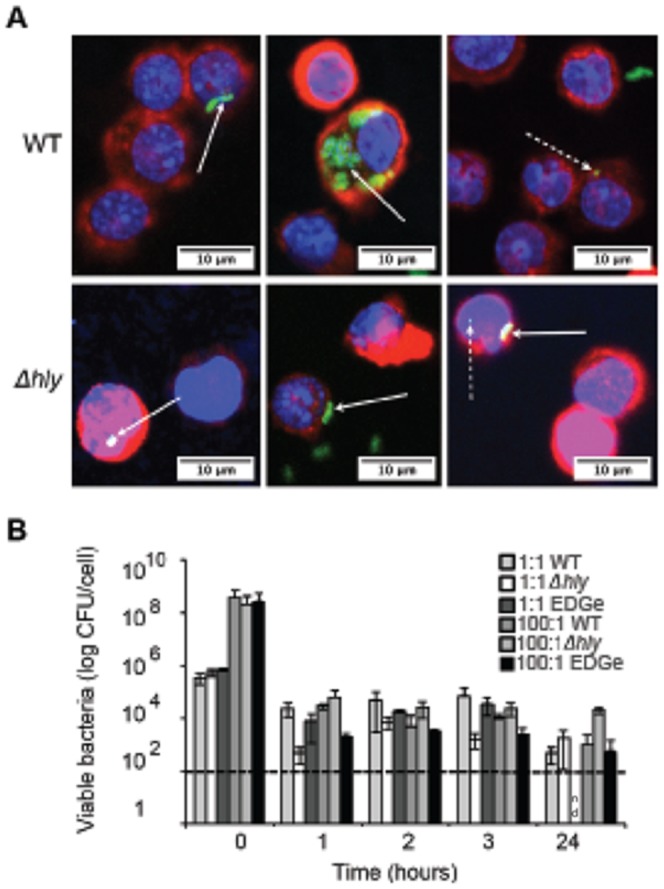
*L. monocytogenes* infects mast cells at low levels and survives intracellularly for up to 24 h. (A) BMMC were incubated with WT or *Δhly* bacteria for 2 h at MOI 10∶1, washed then transferred to fresh media containing 50 µg/ml gentamicin for 24 h. Cells were stained with anti-*Listeria* (green), wheat germ agglutinin (red) and DAPI (blue) and analysed by confocal microscopy. Intracellular whole bacteria (unbroken arrow) and bacterial fragments (broken arrow) are indicated. (B) BMMC were incubated with WT, *Δhly* or EGDe bacteria for 2 h at MOI 1∶1 and 100∶1, washed then transferred to fresh media containing 50 µg/ml gentamicin for the times indicated. At each time point BMMC were washed, lysed and viable intracellular bacteria determined. nd, non detected. Data are (A) representative images of mast cells showing intracellular bacteria from a single experiment; (B) the mean +/- SD from 2 independent experiments.

In an effort to resolve the controversy regarding the ability of *L. monocytogenes* to infect mast cells, we examined the infection of BMMC using two independent assays. One major limitation of the mouse model for *L. monocytogenes* infection is that the bacterial internalin A (InlA) cannot bind to mouse E-cadherin [18]. Since E-cadherin is expressed on mast cells [19] and is potentially important for infection, we used a *L. monocytogenes* strain with a modified InlA, which can bind to murine E-cadherin [20] as the WT strain in this study. As LLO is known to be important for intracellular replication of *L. monocytogenes* [21], [22], we used wild type (WT) and LLO-deficient (*Δhly*) *L. monocytogenes* to investigate the requirements and outcome of mast cell infection. Analysis using confocal microscopy showed that both WT and *Δhly L. monocytogenes* could be detected intracellularly, but only in very few mast cells (Figure 1A). Furthermore, bacterial fragments which were smaller than whole bacteria were identified in 7.3% of the 41 cells analysed in total.

To confirm that the bacteria observed by microscopy were indeed intracellular and to examine the rate of *L. monocytogenes* infection and intracellular survival, BMMC were infected at a multiplicity of infection (MOI) of 1∶1 and 100∶1. Similar numbers of viable intracellular WT and *Δhly* bacteria were recovered up to 24 h after exposure to each MOI (Figure 1B). Bacteria only controls confirmed that extracellular bacteria were killed by the antibiotic treatment (data not shown). Before the addition of antibiotic at T = 0, there was a difference in the total bacterial number, as intracellular and extracellular bacteria could not be distinguished at this point. Interestingly, the MOI did not appear to be a limiting factor for the number of viable intracellular bacteria per mast cell and overall there was no obvious difference in infection rate or intracellular survival of WT or *Δhly* bacteria (Figure 1B). Interestingly, we also observed no difference in the infection rate between the *L. monocytogenes* strains with a modified InlA (WT) and the parental strain, EGDe, which does not bind murine E-cadherin (Fig. 1B). Thus *L. monocytogenes* infects BMMC at low levels and survives intracellularly for up to 24 h in an LLO-independent manner.

### LLO is required for calcium-independent mast cell degranulation

Mast cells are important for resolving *L. monocytogenes* infection *in vivo* [3] but the inefficient infection of mast cells by *L. monocytogenes* is unlikely to account for their importance. *L. monocytogenes* and specifically the pore-forming toxin LLO [23], cause mast cell degranulation *in vivo* [24] and *in vitro* [3]. Mast cells have also been shown to release cytokines directly in response to *L. monocytogenes* under various conditions [6], [7], [9], [10], [24]. We therefore focused on further investigating the effect of *L. monocytogenes* on mast cell activation, specifically mast cell degranulation and mediator release.

**Figure 2:**
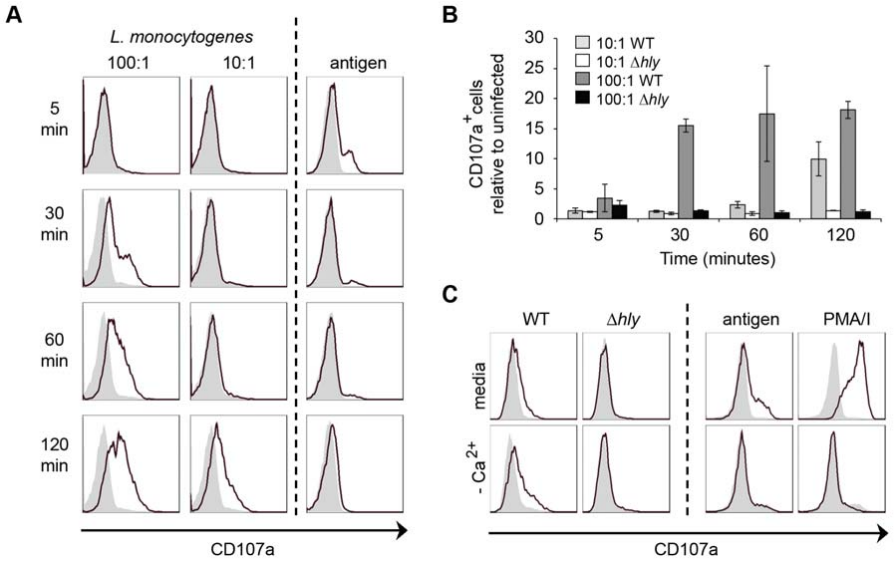
*L. monocytogenes* causes calcium-independent LLO-mediated mast cell degranulation with delayed kinetics. (A and B) BMMC were incubated for the times indicated with (A) media alone (shaded) or WT *L. monocytogenes* at the MOI indicated (black line); IgE-loaded cells were unstimulated (shaded) or stimulated with 10 ng/ml antigen (black line); (B) WT or *Δhly L. monocytogenes* at the MOI indicated. (C) BMMC were incubated for 2 h alone (shaded), or with WT or *Δhly L. monocytogenes* at MOI 100:1 (black line) in media or calcium-free buffer. IgE-loaded cells were stimulated with 10 ng/ml antigen or PMA/I. CD107a expression levels were determined by flow cytometry. Data are (A and C) representative of 3 independent experiments; (B) mean +/- SEM for 3 independent experiments shown relative to media alone.

We examined the kinetics of *L. monocytogenes-*mediated degranulation using surface CD107a expression as a marker. Unlike the rapid and transient antigen-mediated CD107a upregulation, *L. monocytogenes-*mediated CD107a upregulation was delayed and sustained (Figure 2A). The response was further delayed at a lower MOI (Figure 2A and B) and was completely dependent on LLO (Figure 2B and C). Antigen-mediated β-hexosaminidase release is calcium-dependent with complete inhibition in the absence of extracellular calcium [10]. We used calcium-free buffer to determine whether the observed degranulation was a result of calcium-dependent signalling or as a result of the pore-forming activity of LLO. Whilst there was no CD107a upregulation in response to antigen or PMA/I in calcium-free buffer, LLO-mediated CD107a upregulation occurred in the absence of calcium (Figure 2C). *L. monocytogenes*, therefore, causes calcium-independent LLO-mediated mast cell degranulation with delayed kinetics.

### *Listeria monocytogenes* modulates the surface expression of CD117 on mast cells

Two important receptors involved in mast cell survival, proliferation and activation are CD117 (c-kit) and FcεRI [25]. FcεRI is constitutively expressed on mast cells, and antigen-mediated cross-linking of FcεRI specifically causes internalisation of the FcεRI signalling complex [26]. High levels of CD117 are expressed on mast cells, which can also be internalised or shed upon activation [27], [28], [29], [30]. We therefore examined whether *L. monocytogenes*-mediated mast cell activation affects the surface expression of CD117 and FcεRI.

**Figure 3:**
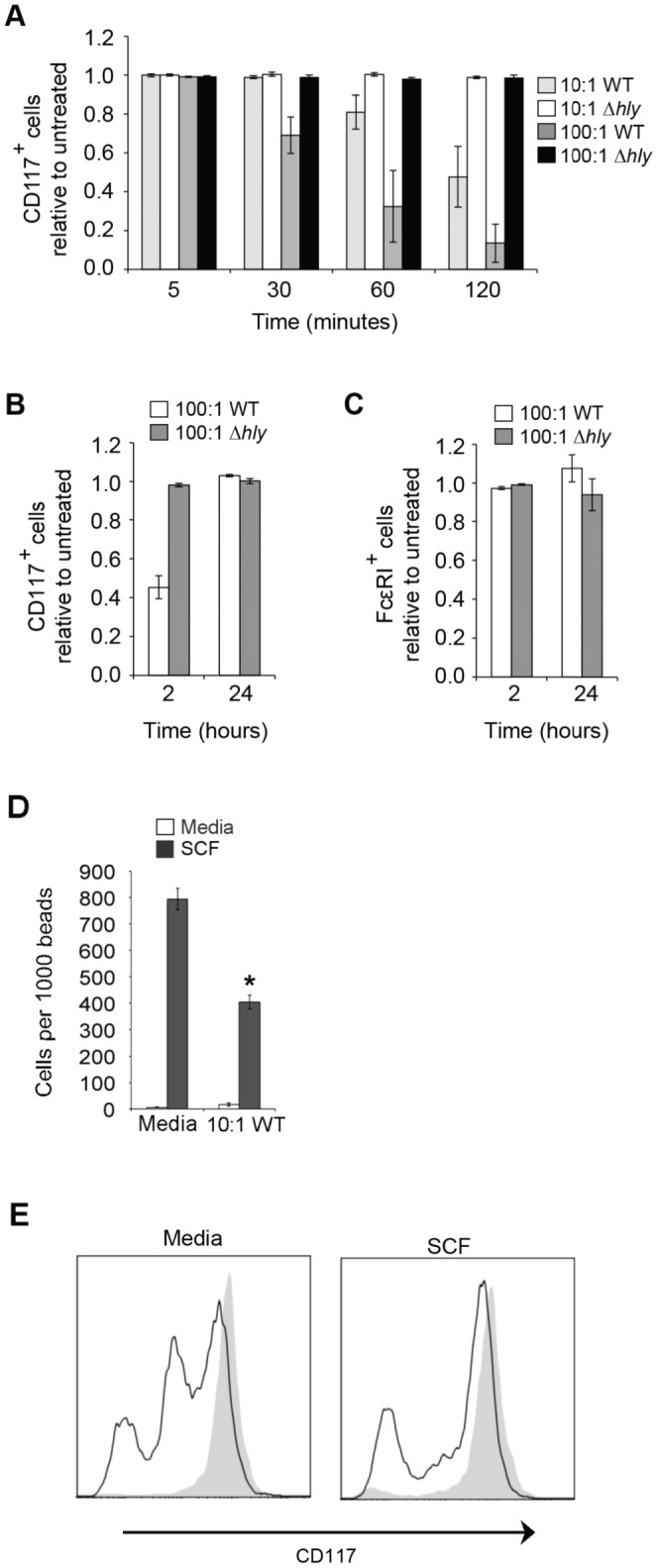
*L. monocytogenes* causes LLO-mediated downregulation of CD117 but not FcεRI on BMMC and decreases chemotaxis. (A, B and C) BMMC were incubated with WT or *Δhly* bacteria at the MOI indicated for (A) the times indicated; (B and C) 2 h, then washed and transferred to media containing 50 µg/ml gentamicin for an additional 24 h. Cells were analysed at the time points indicated. CD117 and FcεRI levels were determined by flow cytometry and are shown relative to untreated cells. (D and E) BMMC were incubated with or without WT bacteria at the MOI 10∶1 for 2 h prior transfer to the top section of a transwell. Cells were incubated in the transwells for 4 h in the presence or absence of 10 ng/ml SCF in the bottom compartment. Migration was assessed by counting number of cells per 1000 beads by flow cytometry (D). (E) CD117 levels were determined by flow cytometry in cells incubated in media alone (shaded) or with 10:1 MOI WT bacteria (Black line) for cells incubated with media or in the presence of 10 ng/ml SCF. Data are the mean +/- SEM for 3 (A, B and C); 7 (D); 4 (E) independent experiments. Statistical significance p<0.05 compared to media treatment is indicated (*).

*L. monocytogenes* caused downregulation of CD117 on the surface of BMMC in an LLO-dependent manner (Figure 3A and B). The peak in downregulation of CD117 coincided with the peak in degranulation measured by CD107a upregulation, and was similarly delayed at a lower MOI (Figure 2B and 3A). The downregulation of CD117 expression was transient, as normal expression levels were detected 24 h after initial exposure to *L. monocytogenes* (Figure 3B). FcεRI expression, however, was unaffected by *L. monocytogenes* treatment (Figure 3C). The functional consequence of the downregulation of CD117 after exposure for 2 h to *L. monocytogenes* was tested in an assay of chemotaxis. The migratory ability of BMMC exposed to *L. monocytogenes* to SCF, the CD117 ligand, was significantly lower than that of uninfected control cells (Figure 3D). The downregulation of the CD117 receptor upon *L. monocytogenes* treatment in the transwell system was assessed by flow cytometry (Figure 3E). However, one cannot exclude the possibility that the difference in the chemotactic ability of the cells is affected by other mechanisms, which are dependent on listeria exposure yet independent of the CD117 downregulation.

*L. monocytogenes*, therefore, selectively alters the surface receptor expression of BMMC in an LLO-dependent manner.

### Mast cells release a wide range of mediators in response to *L. monocytogenes*

Mast cells release β-hexosaminidase [10], [24], TNF-α [6], [24], IL-6 [7], [9], [10] and IL-1β [10] in response to *L. monocytogenes.* As only these few mediators have been investigated, we aimed to better understand the extent of mast cell activation by *L. monocytogenes* by examining a wide range of mediators. We looked at the release of pre-formed mediators after stimulation for 2 h as well as the release of newly synthesised mediators released 24 h later. To allow detection of newly synthesised mediators, after the initial stimulation mast cells were transferred to fresh media containing gentamicin to kill any remaining bacteria.

**Figure 4:**
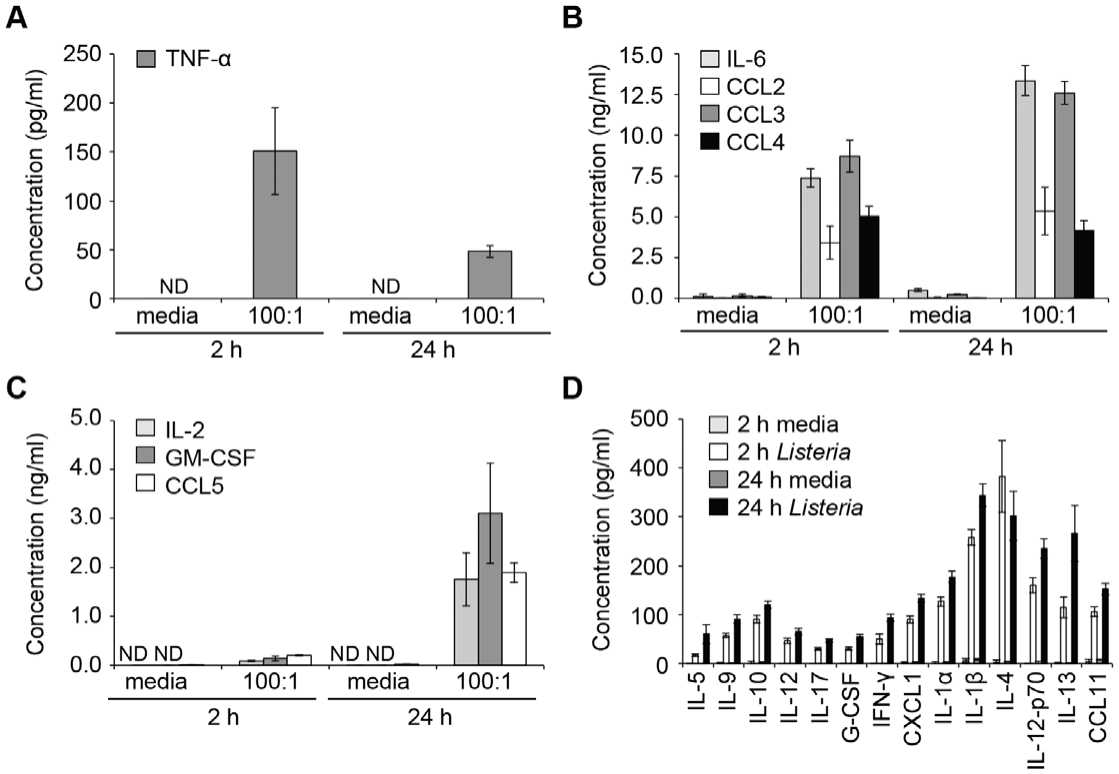
*L. monocytogenes* causes the release of various cytokines and chemokines from BMMC. BMMC were incubated with media alone or WT *L. monocytogenes* at MOI 100:1 for 2 h, washed and transferred to media containing 50 µg/ml gentamicin for an additional 24 h. Cell supernatants were harvested after the initial 2 h and the additional 24 h and mediator release determined by Bio-plex. Concentrations of (A) TNF-α; (B) IL-6 (light grey bars), CCL2 (open bars), CCL3 (dark grey bars) and CCL4 (black bars); (C) IL-2 (light grey bars), GM-CSF (dark grey bars) and CCL5 (open bars); (D) the mediators indicated at 2 h and 24 h in media alone (light grey bars and dark grey bars, respectively) or with *Listeria* (open bars and black bars, respectively). Undetectable levels are indicated (ND). Data are mean +/- SEM for 4 independent experiments.

Early release of TNF-α was observed with only low levels of secretion of newly synthesised TNF-α during the 24 h without bacteria (Figure 4A). The timing of TNF-α release, therefore, coincided with mast cell degranulation (Figure 2A and B). In contrast, IL-6 together with CCL2, CCL3 and CCL4 showed early release in response to *L. monocytogenes* but their secretion was sustained, and in some cases increased after 24 h (Figure 4B). There was no early release of IL-2, GM-CSF and CCL5, however, but they were secreted at 24 h (Figure 4C). In addition to these key mediators, other mediators were released in lower quantities (Figure 4D). *L. monocytogenes*, therefore, causes the secretion of a wide range of mediators from mast cells, with the kinetics of release varying depending on the specific mediator.

### Listeria interaction with mast cell E-cadherin affects IL-6 and CCL2 release

**Figure 5:**
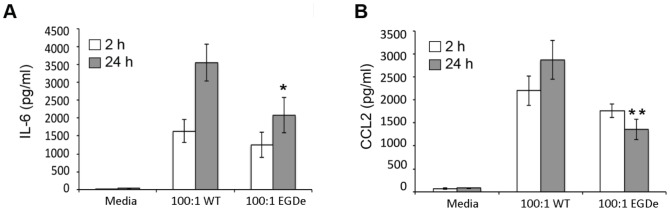
*L. monocytogenes* strain with a modified InlA causes the release of higher concentration of IL-6 and CCL2. (A and B) BMMC were incubated with media alone, WT or EGDe *L. monocytogenes* at the MOI indicated for 2 h, washed and transferred to media containing 50 µg/ml gentamicin for an additional 24 h. Cell supernatants were harvested after the initial 2 h (open bars) and the additional 24 h (grey bars). (A) IL-6 and (B) CCL2 concentrations were determined by ELISA. Data are mean +/- SEM. Statistical significance between WT and EGDe treated at p<0.05 (*) and p<0.01 (**) is indicated.

As discussed, *L. monocytogenes* internalin A (InlA) binds to human but not mouse E-cadherin [18] and since E-cadherin is expressed on mast cells [19] we have used a *L. monocytogenes* strain with a modified InlA, which can bind to murine E-cadherin [20], as the WT strain in this study. In order to determine whether the InlA interaction with E-cadherin was playing a role in the mast cell IL-6 and CCL2 production in response to *L. monocytogenes,* BMMC were incubated with our WT strain or the parent EGDe strain, with unmodified InlA. Mast cells produced higher levels of IL-6 in response to infection with the *L. monocytogenes* strain with a modified InlA (Figure 5A and 5B) suggesting that the interaction with E-cadherin potentiates cytokine and chemokine release.

### LLO is required for early mediator release from mast cells

**Figure 6:**
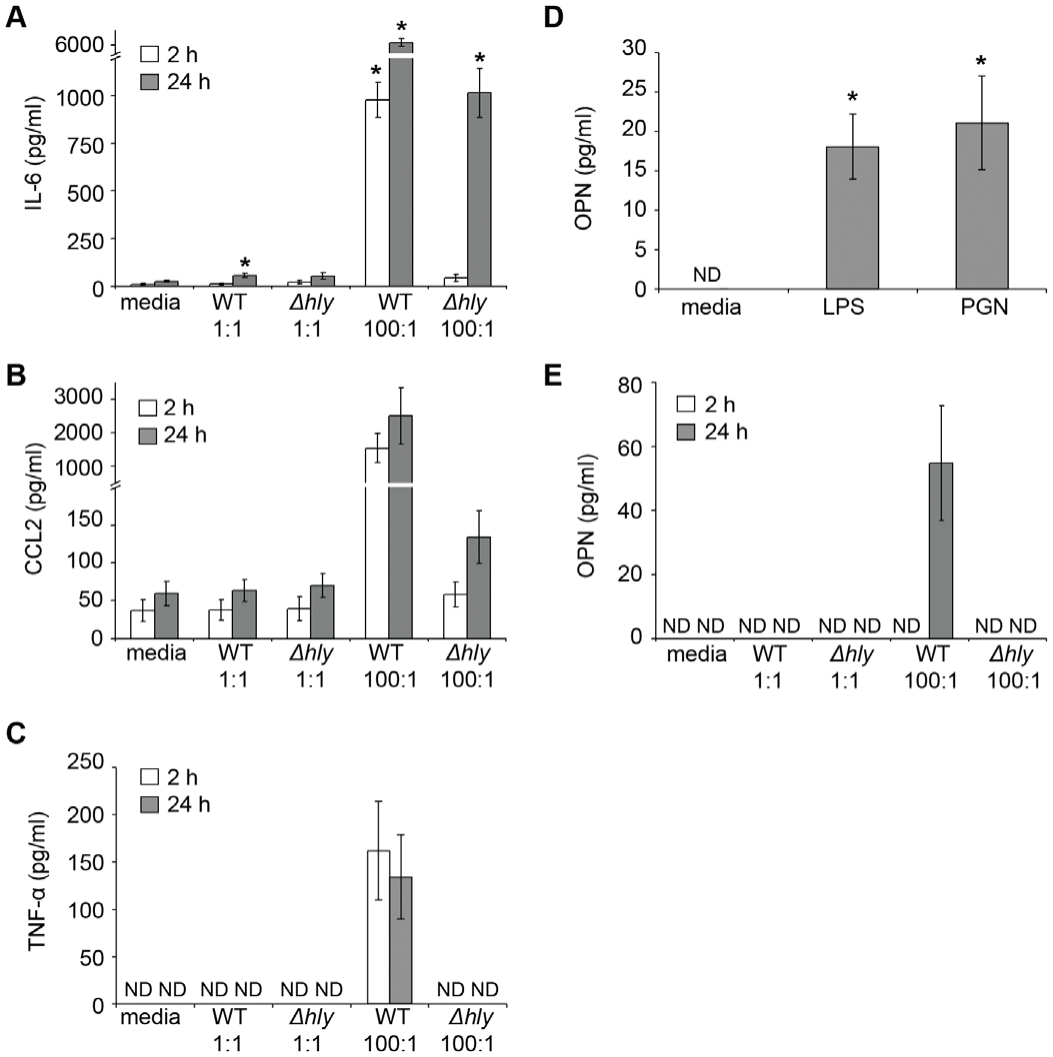
BMMC show mediator release in response to bacterial PAMPs and *L. monocytogenes* in an LLO-dependent manner. (A, B, C, D and F) BMMC were incubated with media alone, WT or *Δhly L. monocytogenes* at the MOI indicated for 2 h, washed and transferred to media containing 50 µg/ml gentamicin for an additional 24 h. Cell supernatants were harvested after the initial 2 h (open bars) and the additional 24 h (grey bars). (E) BMMC were incubated with media alone, 100 ng/ml LPS or 10 µg/ml PGN for 24 h. (B) IL-6, (C) CCL2, (D) TNF-α, and (E and F) OPN concentrations were determined by ELISA, with values below the limit of detection indicated (ND). Data are mean +/- SEM for (B) 5, (C) 4, (D) 4, (E) 8, and (F) 3 independent experiments. Statistical significance p<0.05 compared to media treatment is indicated (*).

TNF-α release from BMMC is known to be LLO-dependent to some extent, although *Δhly L. monocytogenes* still causes its secretion [6]. IL-1β release *in vivo*, however, is LLO-independent [6] but it is unclear whether this is a result of secretion by mast cells. We further examined the requirement of LLO for mediator release from BMMC. IL-6 and CCL2 were used as a representative cytokine and chemokinedue to their potent early and sustained release in response to *L. monocytogenes* (Figure 4B). TNF-α release was also examined due to its importance as a pre-formed mast cell mediator. The release of IL-6, CCL2 and TNF-α was MOI-dependent, with greater secretion at MOI 100∶1 than at MOI 1∶1 in response to both WT and *Δhly* bacteria (Figure 6A, B, C). LLO was required for early release of IL-6 and for both the early and sustained release of CCL2 and TNF-α (Figure 6A-C). In the absence of LLO, however, BMMC still showed delayed release of IL-6 and CCL2, but not of TNF-α (Figure 6A, B, C).

OPN is known to be crucial to clear *L. monocytogenes* infections *in vivo* [12] and BMMC have been shown to release it upon antigen-mediated degranulation [15]. We examined the release of OPN from BMMC stimulated with the bacterial pattern-associated molecular patterns (PAMPs) lipopolysaccharide (LPS) and peptidoglycan (PGN). As both LPS and PGN caused OPN release from mast cells (Figure 6D) and LLO is known to be a TLR4 agonist [31], we examined OPN release in response to WT or *Δhly L. monocytogenes.* Release of OPN was also observed 24 h after *L. monocytogenes* exposure, but only with high titres of WT bacteria (Figure 6E). LLO is therefore required for early mediator release from mast cells though it is not absolutely required for the later release of some mediators such as IL-6.

### MyD88 is required for IL-6 and CCL2 release

**Figure 7:**
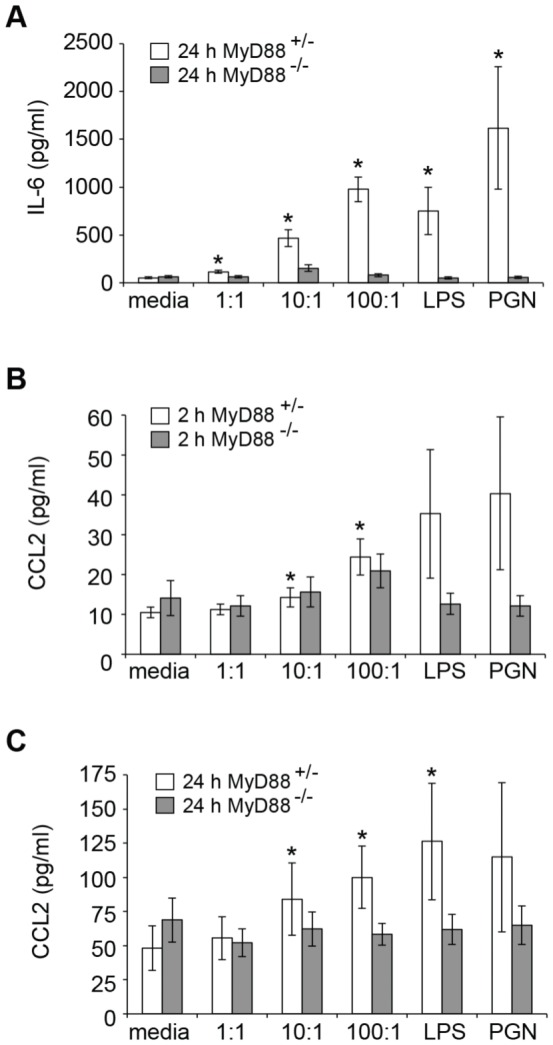
IL-6 and CCL2 secretion in response to *L. monocytogenes* is MyD88-dependent. BMMC from MyD88^+/-^ and MyD88^-/-^ mice were incubated with media alone, WT *L. monocytogenes* at the MOI indicated, 100 ng/ml LPS or 100 µg/ml PGN for 2 h, washed and transferred to media containing 50 µg/ml gentamicin for an additional 24 h. Cell supernatants were harvested at the times indicated for MyD88^+/-^ (open bars) and MyD88^-/-^ (grey bars), and (A) IL-6 and (B and C) CCL2 levels were determined by ELISA. Data are mean +/- SEM for (A) 6; (B and C) 5 independent experiments. Statistical significance p<0.05 compared to media treatment is indicated (*).

The adaptor protein MyD88 is critical for several TLR signalling pathways [32]. MyD88 is required to some extent for TNF-α secretion from BMMC in response to *L. monocytogenes in vitro* but is not required for IL-1β secretion *in vivo* [6]. To further examine the importance of MyD88 for mediator release, we exposed BMMC derived from MyD88^+/-^ and MyD88^-/-^ mice to WT *L. monocytogenes*. In the absence of WT littermate controls, MyD88^+/-^ BMMC were used as controls, although they had reduced MyD88 protein levels compared to WT BMMC (data not shown). Mediator release from MyD88^+/-^ BMMC (Figure 7) in general was reduced compared to WT BMMC (Figure 6), possibly due to the reduced MyD88 levels.

The release of IL-6 in response to *L. monocytogenes* and bacterial PAMPs was prevented in the absence of MyD88 (Figure 7A). MyD88 was required for both the early (Figure 7B) and late (Figure 7C) release of CCL2 in response to intermediate and high titres of bacteria. Mediator release in response to WT *L. monocytogenes* is therefore dependent on MyD88 expression.

## Discussion

In this study we examined three key aspects of mast cell biology in response to *L. monocytogenes*: mast cell infection, alteration of mast cell phenotype, and mediator release. We demonstrate that mast cells are infected by *L. monocytogenes in vitro,* but that this infection in inefficient compared to the infection of other leukocytes such as macrophages and dendritic cells [33]. This inefficiency cannot be explained by bacterial titre as 100-fold increase in initial bacterial numbers has no effect on the number of intracellular bacteria suggesting that a property of mast cells limits the rate of infection. Mast cell infection is LLO-independent, which is surprising as LLO is important for intracellular survival by mediating bacterial escape from the phagosome in both phagocytic and non-phagocytic cells [4].

Our data on infection of mast cells by *L. monocytogenes* confirm the low level uptake *in vivo* [3] and *in vitro* [17], but contradict other *in vitro* studies where BMMC infection was not observed [16], [24]. Two key differences which could explain this are the bacterial strain and methods used in the studies. Firstly, we used *L. monocytogenes* with a modified internalin A, which binds murine E-cadherin [20], unlike WT *L. monocytogenes* [18]. Unlike macrophages, mast cells are not professional phagocytes, so in the absence of complement the main route of bacterial entry would be via E-cadherin-InlA internalisation [4] and BMMC are known to express E-cadherin [19]. During *in vitro* infection of BMMC with *L. monocytogenes* in the absence of complement, the choice of strain therefore could be crucial and WT strains lacking this modified InlA would not be expected to infect mast cells via E-cadherin. Surprisingly, we find that the presence of the interaction with E-cadherin does not affect the infection rate but modulates slightly the IL-6 release and in a more pronounced manner the CCL2 secretion. Thus this interaction may affect the outcome of mast cell exposure to *Listeria* such as mediator release and mast cell degranulation.

Secondly, we analysed infection using two independent methods: microscopy and recovery of viable intracellular bacteria, as the latter method has been used to successfully identify intracellular *L. monocytogenes* following mast cell infection *in vitro* [17]. By determining viable intracellular bacteria in addition to analysis by microscopy we were able to screen large numbers of cells to confirm our findings.

Analysis of mast cell infection by microscopy revealed that in addition to whole intracellular bacteria, some cells contained smaller bacterial fragments, which may be the remains of bacteria digested intracellularly. As these fragments were detected with both WT and *Δhly L. monocytogenes*, it cannot be due to LLO-mediated bacterial escape from the phagosome [4]. Mast cells have anti-microbial activities involving the generation of superoxide anions [34] and contain proteases in their granules [1], so have the potential to kill and digest intracellular bacteria. It is unlikely that the bacteria were killed and digested extracellularly then phagocytosed, as we show that *Δhly L. monocytogenes* did not induce mast cell degranulation under these conditions, and would therefore not induce the release of proteases. We therefore suggest that mast cells may be capable of killing and digesting intracellular *L. monocytogenes* in an LLO-independent manner, although the significance of this observation requires further investigation.

Previous *in vitro* analysis of mast cells and *L. monocytogenes* focused on mast cell activation in response to the bacteria [6], [16], [24], so to add to these findings, we examined changes to mast cell phenotype. To our knowledge, we provide the first evidence that *L. monocytogenes* alters mast cell phenotype in an LLO-dependent manner. The expression of CD117 on mast cells decreases over time in the presence of viable WT but not *Δhly L. monocytogenes*. This could be caused by bacterial growth and the subsequent increase in LLO concentration over time. When mast cells were transferred to fresh media and extracellular bacteria killed, CD117 expression levels returned to normal, demonstrating this downregulation is reversible. It therefore would be interesting to examine the effect of recombinant LLO on CD117 expression, which would also allow the effects of prolonged exposure to be investigated. FcεRI expression levels, in contrast, remain unaltered after exposure to *L. monocytogenes.* This is interesting as the FcεRI signalling complex is internalised upon antigen-mediated degranulation [26], and further illustrates the difference between antigen and LLO-mediated degranulation. It is possible that the presence of IgE may be required for FcεRI internalisation in response to *L. monocytogenes.*

CD117 is the receptor for SCF. Therefore, upon downregulation of the receptor mast cells would be expected to be less responsive to SCF. The expression of CD117 on mast cells is tightly regulated [35] and our finding that *L. monocytogenes* induces a transient decrease adds to the known mechanisms of receptor downregulation such as activation of the receptor by SCF or other cytokines [35]. Mast cells constitutively express high levels of CD117 and signalling through the receptor is important for mast cell migration, survival and proliferation [25]. Indeed, we show that *L. monocytogenes* infection of BMMC affects their chemotactic activity in response to SCF. However, preliminary data from our laboratory show that the LLO-deficient *L. monocytogenes* mutant (*Δhly)*, which does not induce BMMC degranulation and CD117 downregulation, also impairs BMMC migration to SCF. These findings suggest that exposure to *L. monocytogenes* affects SCF-driven cell migration independently from the level of CD117 expression.

CD117 is also important for mast cell activation [25] and SCF is known to enhance antigen-mediated degranulation [36].

Furthermore, CD117 expression is required for the maximal response to IL-33 [37]. The first stage of *L. monocytogenes* infection involves crossing the intestinal barrier [4] and IL-33 is released upon epithelial damage [38], so presumably IL-33 is released during infection. Mast cells respond potently to IL-33 [39], and reduced IL-33-mediated mast cell activation, as a consequence of reduced CD117 expression [37], could contribute to pathology *in vivo. L. monocytogenes*-mediated CD117 downregulation, therefore, may have profound effects on mast cell survival, proliferation and activation.

The loss of CD117 could be due to either receptor internalisation or shedding. Receptor internalisation and degradation, similar to that seen when CD117 binds SCF, is one possible explanation [30]. This is dependent on both phosphatidylinositol 3- (PI 3-) kinase activation and Ca^2+^ influx [28], and LLO has been shown to induce both transient protein tyrosine phosphorylation and Ca^2+^ influx in mast cells [24]. CD117 shedding by tumour necrosis factor α-converting enzyme (TACE) is an alternative explanation for the CD117 downregulation we observed [29]. We cannot be certain if either of these two mechanisms is predominant or whether both are involved, and this is a matter for further investigation. Irrespective of the mechanism of CD117 downregulation, we would suggest that overall the consequences for mast cell survival, proliferation and activation would be similar.

Previous studies have examined mast cell degranulation in response to *L. monocytogenes* and we investigated this further using antigen-mediated degranulation for comparison. *L. monocytogenes* causes LLO-dependent mast cell degranulation which is calcium independent [24], and our data confirm these findings. Contrary to this study, we found that in the absence of calcium LLO-mediated degranulation was enhanced, whereas antigen- and PMA/I-mediated degranulation were completely inhibited. This contradiction could be explained by the use of different media. As LLO is a pore-forming toxin [23] known to damage intracellular compartments such as the endoplasmic reticulum [24], it is possible that LLO forms pores in granules resulting in degranulation and mediator release. This could explain the calcium-independent nature of LLO-mediated degranulation. Calcium-dependence is just one key difference between antigen- and LLO-mediated mast cell degranulation. There are clear differences in the kinetics of degranulation with antigen-mediated degranulation being rapid and transient, whereas LLO-mediated degranulation is delayed and sustained. This difference could be explained by bacterial growth and an increase in LLO concentration over time.

Degranulation and β-hexosaminidase release has been shown to be LLO-dependent, and it has been inferred that TNF-α secretion is therefore also LLO-dependent [24]. We demonstrate that the early release of the pre-formed cytokines TNF-α and IL-6 is LLO-dependent, and that this release coincides with degranulation. The delayed release of IL-6 but not TNF-α is LLO-independent, contradicting previous reports of LLO-independent TNF-α release in response to *L. monocytogenes* [6]. There are, however, methodological differences between our studies and the shorter incubation times of mast cells with bacteria could account for the comparatively low levels of TNF-α observed in this study. The delayed release of IL-6 could be due to TLR signalling [32], with TLR2 being an obvious candidate, as it is required for the LLO-independent induction of TNF-α transcripts [6].

IL-6, IL-1 and TNF-α are important cytokines in the immune response against *Listeria* [40], [41], [42], therefore their production by mast cells upon exposure to the bacteria may contribute to the immune defence against this infection. Upon *L. monocytogenes* exposure, BMMC also secreted the T cell growth factor IL-2 but did not produce a cytokine profile that would be expected to particularly skew the T cell response.

Mast cells produced GM-CSF in response to *L. monocytogenes*, a growth factor which has previously been shown to be required for the immune response to the bacteria [43]. When infected with *L. monocytogenes*, GM-CSF^-/-^ animals exhibited higher bacterial loads and reduced macrophage recruitment [43]. This phenotype is thought to be due to a defect in haematopoiesis in the absence of the growth factor [43]. Recently, however, it has been reported that Csf-2r-deficient (Csf2rb^−/−^Csf2rb2^−/−^) mice that lack the common β subunit (Csf2rb) and the IL-3r unique β subunit (Csf2rb2) were equally efficient as WT mice in controlling *L. monocytogenes* bacterial burden in the spleen and liver early after infection [44]. Whether this discrepancy is due to the use of different mouse strains or to the deletion of the IL-3 signalling receptor is unclear. Therefore, mast cell-derived GM-CSF may act during an immune response to promote the growth of progenitors in the bone marrow to ensure that sufficient numbers of macrophages are available to fight the infection though the importance of this remains unclear.

In addition to the release of cytokines, we also demonstrate that mast cells release various chemokines in response to *L. monocytogenes*. As with IL-6, the early release of CCL2 is LLO-dependent and the late release is LLO-independent. It seems, therefore, that LLO is required to induce the release of preformed mediators from mast cells, in agreement with the requirement for the toxin to cause degranulation. The early release of large quantities of chemokines including CCL2, CCL3 and CCL4 suggests that they may be pre-stored in mast cells. This is particularly interesting since, to our knowledge, chemokines have not previously been described as pre-formed mast cell mediators [45].

Chemokines have previously been shown to be important in infection and their release by mast cells could contribute to immune defence against bacteria. Upon *L. monocytogenes* infection, CCR2^-/-^ animals show defective macrophage recruitment and are unable to clear the bacteria as effectively as WT mice [46]. We have shown that mast cells produce a CCR2 ligand, CCL2, upon exposure to *L. monocytogenes* and this chemokine may therefore play a role in macrophage recruitment *in vivo*. There is considerable redundancy in the chemokine receptor system [47], for example CCL3, CCL4 and CCL5, three chemokines which we have found to be produced by mast cells upon *L. monocytogenes* challenge, all bind CCR5 with both CCL3 and 5 additionally binding CCR5 and CCL5 binding CCR3 [47]. It is therefore interesting to note that CCR5^-/-^ mice have been shown to respond to *L. monocytogenes* in a similar manner to WT and are not defective in bacterial clearance [48]. It may be that in the absence of CCR5 other chemoattractant receptors such as CCR1 act to retain cell recruitment.

In addition to their role in leukocyte recruitment, CCL3, 4 and 5 have been shown to activate macrophages in an *in vitro* model of *L. monocytogenes* infection [49] suggesting that mast cell production of these chemokines may assist in the immune defence against the bacteria by activating macrophages.

MyD88 is an important signalling molecule involved in many TLR signalling pathways in mast cells [32] and is important for TNF-α release in response to *L. monocytogenes* [6]. We show that IL-6 and CCL2 release in response to *L. monocytogenes* is MyD88-dependent, indicating that cytokine and chemokine release may result from the activation of similar signalling pathways.

OPN has been shown to be a key mediator during the immune response against *L. monocytogenes* in one study [12] whilst a later study found that WT and OPN^-/-^ mice were equally susceptible to the bacteria [13]. The discrepancies between these two studies could be due to several factors. In the original study, the OPN^-/-^ mice were on a mixed 129/C57BL/6 background whereas in the later work the animals were backcrossed onto a C57BL/6 background [12], [13]. The difference in background is particularly important since C57BL/6 animals have been shown to be resistant to *L. monocytogenes* infection whereas 129 animals are more susceptible [50]. In addition, in the study which found no role for OPN, the animals were infected with almost 10 fold more bacteria than in the original paper [12], [13]. It may therefore be the case that OPN has a greater role to play in an infection with a lower number of bacteria, or that the importance of OPN in the immune response to *L. monocytogenes* may depend on the genetic susceptibility of the host [11]. Further work is needed to fully elucidate the role of OPN during an *in vivo* infection, but OPN does play an important role under certain circumstances [12] and is a well described immunomodulator [11].

Here, we show that OPN is released by mast cells in response to *L. monocytogenes* in an LLO dependent manner. Although OPN is known to be released by T cells [14], the source during *L. monocytogenes* infection is unknown. Here we show that mast cells release OPN within 24 h of contact with *L. monocytogenes* and propose that mast cells are a potential source of OPN during the early stages of infection.

Th1 responses are important during *L. monocytogenes* infection [51] and LLO specifically can inhibit Th2 responses by skewing Th1 cell differentiation [52]. Indeed, BTBR mice, with their predominant Th2 profile, have increased bacterial burdens in the liver and spleen following infection [53]. As mast cells are known to release the pro-inflammatory cytokines TNF-α, IL-6 and IL-1β in response to *L. monocytogenes* [6], [7], [9], [10], [24] we expected they might, therefore, release a range of Th1 cytokines. Interestingly, typical Th2 cytokines including IL-4 and IL-13 were released in response to *L. monocytogenes.* This would suggest that LLO may induce general cytokine secretion from mast cells and that other cells may also be involved in determining the effector response.

In summary, we addressed the controversy of mast cell infection by *L. monocytogenes* and here we demonstrate mast cell infection and intracellular survival of *L. monocytogenes* is LLO-independent. We add to previous findings by showing that LLO-mediated mast cell activation causes release of pre-formed and newly synthesised chemokines and OPN in addition to cytokines. We propose that the importance of mast cells during *L. monocytogenes* infection *in vivo* could stem from the wide range of mediators they release, allowing the rapid initiation of the immune response. Although these mediators may coordinate the recruitment and activation of leukocytes, it is unlikely that the initial response of mast cells to *L. monocytogenes* favours the development of the Th1 responses required to resolve infection.

## Materials and Methods

### Mice

C57BL/6 mice were housed in full accordance with the Animal Scientific Procedures Act 1986 under Home Office approval. In consensus with the United Kingdom Animal (Scientific Procedures) Act of 1986, this study did not require a Home Office project license because no regulated procedures were carried out. *MyD88^+/-^* and *MyD88^-/-^* mice were housed in accordance with institutional guidelines, and all experimental procedures were approved by the institutional animal care and use committee of the Research Center Borstel (Ministry of Agriculture, the Environment, and Rural Areas, Schleswig-Holstein).

### BMMC differentiation and culture

BMMC were differentiated from bone marrow isolated from the femurs of mice. Cells were cultured for 4 weeks in Iscove’s modified Dulbecco medium (IMDM) supplemented with penicillin, streptomycin, L-glutamine (both from PAA), 10% heat-inactivated foetal bovine serum (Biochrom), β-mercaptoethanol, non-essential amino acids, sodium pyruvate, and vitamins (all from Invitrogen) at 37°C with 7.5% CO_2_ with 10 ng/ml SCF and 5 ng/ml IL-3 (both from R&D Systems). After 4 weeks cells were analysed by flow cytometry and >90% were c-kit^+^(CD117)FcεRI^+^ST2^+^. Cells were cultured for a further 4 weeks at 0.5-1×10^6^ cells/ml.

### Bacterial cultures

*Listeria monocytogenes* strain EGDe, EGDe InlA^m^ (termed WT) [20], and its *Δhly* derivative [54] were used. Cells were cultured in tryptone soya broth (TSB) or agar (Fisher) at 37°C. Glycerol stocks were prepared in PBS-15% glycerol, using bacterial cultures with absorbance at 600 nm ∼ 0.5. Aliquots were frozen in liquid nitrogen then stored at -80°C and viable cell numbers determined.

### BMMC co-culture with *L. monocytogenes*

Co-culture experiments were performed in BMMC media without streptomycin or penicillin unless otherwise stated. Where stated, calcium-free buffer (PBS without calcium/magnesium, 1% BSA, 4.5 mg/ml D-glucose (all from Sigma)) was used. For incubations longer than 4 h, media contained 1 ng/ml IL-3. Where indicated, penicillin, streptomycin and 50 µg/ml gentamycin (Sigma-Aldrich) were included. In order to count intracellular bacteria, BMMC were washed in PBS then lysed in 0.5% Triton X in PBS and CFU determined.

### Fluorescence microscopy

BMMC were seeded onto cover slips using poly-L-lysine (Sigma). Cells were stained with rat anti-*Listeria* polyclonal antibody (Abcam), Goat anti-rabbit IgG Alexa Fluor® 488 F(ab')2, wheat germ agglutinin Alexa Fluor® 555 conjugate (both from Life Technologies) and VECTASHIELD® HardSet™ with DAPI (Vector Laboratories). Images were analysed by confocal microscopy using a Nikon C1 confocal on an upright 90i microscope with a 60x objective and 5x confocal zoom. DAPI, Alexa Fluor® 488 and Alexa Fluor® 555 were excited at 405 nm, 488 nm and 543 nm.

### Cell stimulation, ELISA and Bio-plex

BMMC were stimulated in fresh media containing 1 ng/ml IL-3 at a density of 1×10^6^ cells/ml. Cells were treated with LPS from *Escherichia coli* 0127:B8, PGN from *Staphylococcus aureus*, 100 ng/ml phorbol 12-myristate 13-acetate with 1 μM ionomycin (PMA/I; all from Sigma-Aldrich). Prior to treatment with human-dinitrophenyl albumin (DNP) cells were incubated overnight with 200 ng/ml anti-dinitrophyl IgE (SPE-7; Sigma-Aldrich).

Enzyme-linked immunosorbent assays (ELISAs) were performed on supernatants to determine cytokine concentrations using the antibodies and protein standards from R&D Systems according to their protocols. The Bio-plex (Bio-Rad) system was used according to the manufacturer’s protocols. For experiments including bacteria, all supernatants were filtered through a 0.2 µm filter prior to analysis.

### Chemotaxis assay

BMMC were cultured in the absence of SCF for 3-7 days. Cells were incubated at 37°C with 10% CO_2_ at a density of 1×10^6^ cells/ml with or without WT bacteria for 2 h in media without streptomycin or penicillin containing 1 ng/ml IL-3 then centrifuged at 400 g for 5 minutes prior to transfer to the transwell. 3×10^5^ cells were added to the top compartment of a transwell with 8.0 µm pores (Corning Life Sciences) and were incubated for 4 h in the presence or absence of 10 ng/ml SCF. Migration was determined by counting the number of c-kit^+^(CD117) FcεRI^+^ST2^+^ cells per 1000 counting beads (eBiosciences) by flow cytometry.

### Flow cytometry

Cells were stained in staining buffer: 2% newborn calf serum (Invitrogen), 0.1% sodium azide (Fisons), 0.2 mM EDTA in PBS (Invitrogen). Fc receptors were blocked with anti-CD16/CD32 (eBioscience). Antibodies against c-kit (CD117, clone 2B8; BD Biosciences and eBioscience), T1/ST2 (MD Biosciences), FcεRI, LAMP1 (CD107a), anti-*Listeria* polyclonal antibody (Abcam), anti-rabbit IgG F(ab')2 and the 7-AAD viability stain (both from eBioscience) were used. Fluorescence was detected using an LSRII flow cytometer (BD Biosciences) and data analysed using FlowJo software (version 7.6.1; TreeStar).

### Statistical analysis

Statistical analysis was performed in SPSS (version 19, IBM) using the Wilcoxon signed ranks test. Alternatively, Graphpad prism (version 5.02, Graphpad inc.) was used to perform a Mann-Whitney U test for non-parametric data and one-tailed paired t-tests for parametric data where indicated. Statistical significance of p<0.05 or p<0.01 is indicated by * and ** respectively.
